# Psychometric properties of the empathy questionnaire for children and adolescents in a sample of Tanzanian adolescents

**DOI:** 10.3389/fpsyg.2022.981967

**Published:** 2022-10-06

**Authors:** Megan Cherewick, Ronald E. Dahl, Jenn A. Leiferman, Emily Hipp, Sarah Schmiege

**Affiliations:** ^1^Department of Community and Behavioral Health, Colorado School of Public Health, Aurora, CO, United States; ^2^Institute of Human Development, University of California, Berkeley, Berkeley, CA, United States; ^3^Department of Biostatistics and Informatics, Colorado School of Public Health, Aurora, CO, United States

**Keywords:** empathy, validation questionnaire, Tanzania, adolescence, prosocial, behavioral empathy, confirmatory factor analysis

## Abstract

Construct definitions of empathy have sought to distinguish between different domains of empathetic capacity that are related to psychological distress or wellbeing. This study aims to validate the psychometric properties of the Empathy Questionnaire for Children and Adolescents (EmQue-CA) and to test for measurement invariance by gender in a sample of 579 very young adolescents (270 boys and 309 girls) ages 9-12 from Tanzania. Exploratory and confirmatory factor analysis were completed to assess the factor analytic structure of the EmQue-CA, indicating a three-factor model fit these data well. Concurrent validity was demonstrated through strong significant correlations with prosocial behavior and generosity measures. Convergent validity indicated the behavioral subdimension of empathy, intent to comfort, was significantly and negatively associated with externalizing behaviors. Measurement invariance by gender was not supported for these data due to configural invariance in covariance between cognitive empathy and intent to comfort latent constructs. These findings confirm the EmQue-CA is an important measure of three dimensions of empathy; affective empathy, cognitive empathy, and behavioral empathy (intent to comfort) in a sample of Tanzanian adolescents.

## Introduction

Empathy has been historically defined as a desirable character trait representing the affective and moral capacity to understand emotional states of others (Batson et al., 1987). A review of empathy measures in children and adolescents by Sesso et al., 2021, found that most empathy measures have been shown to be multi-dimensional, with affective and cognitive subdimensions most often cited in validation studies (Sesso et al., 2021). Researchers have also sought to distinguish between sympathy, empathy, and compassion. Sympathy has been equated with affective empathy, an emotionally reactive response from the individual observing distress, while cognitive empathy is the capacity to intellectually understand another person’s experience and appraise and identify with emotional states (Lawrence et al., 2004, Schonert-Reichl et al., 2012, Van der Graaff et al., 2018). Others argue cognitive empathy refers to the capacity to understand another person’s emotions over the threshold of feeling sympathy and instead experiencing internal affective states of others (Clark et al., 2019).

Prior to the introduction of the Empathy Questionnaire for Children and Adolescents, most empathy scales primarily focused on affective and cognitive dimensions (Overgaauw et al., 2017). The EmQue-CA provides a scale that captures a third dimension of empathy, intent to comfort, that measures “the extent to which the child/adolescent is inclined to actually help or support the suffering person” in addition to cognitive and affective empathy (Overgaauw et al., 2017). More recently, studies of empathy have considered how ‘intention to comfort’ through compassionate actions differentiates from both affective and cognitive empathy and encompasses behavioral, conscious decision making to practice a prosocial action to alleviate distress (Singer and Klimecki, 2014). Previous validation of the EmQue-CA tested in China highlighted the association between the empathy dimension *intention to comfort* and behavioral empathy, representing verbal and non-verbal behaviors of empathetic capacity (Lin et al., 2021). Based on the use of intention to comfort to assess behavioral empathy in existing research, these terms will be used interchangeably to highlight the behavioral aspect of empathy that the intention to comfort dimension captures. Behavioral empathy is aligned with the construct of compassion, that goes beyond the capacity to feel (affective empathy) and think (cognitive empathy) about another person’s distress and instead includes the motivation to actively alleviate someone else’s distress through intentional actions (Singer and Klimecki, 2014). Recent emerging research has also suggested that positive empathy may be important to advance empathy research. Positive empathy and the capacity to share joy and happiness experienced by others may be an important counterpoint to understanding empathic capacity of individuals. Contrary to the embodiment of other people’s distress, positive empathy refers to the capacity to share joy and happiness experienced by others (Morelli et al., 2015). Another emerging area of empathy research has focused on extension of empathy to the self, reflected by self-compassion, a protective asset that is related to empathy. Self-compassion has gained increasing attention as a protective asset with greater self-compassion levels associated with significantly lower measures of psychopathology (MacBeth and Gumley, 2012).

The developmental science of adolescence seeks to enhance precision with which we understand the dynamic maturational period from the onset of puberty to adulthood. Empathy is a critical factor in shaping social interactions, and is especially important during adolescence. Differentiating empathy by affective, behavioral, and cognitive dimensions has been recognized as important to uncovering a more nuanced understanding of how empathy is associated with both negative and positive psychological outcomes. In adolescent research, capacity for empathy has been considered integral to social and moral development (Sesso et al., 2021). Research examining opportunities to modify empathetic capacity have identified the close associations between empathy and prosocial behavior (Jolliffe and Farrington, 2006). Higher levels of empathy have been associated with prosocial behaviors, higher levels of emotional regulation and lower aggressive behaviors (Mehrabian and Epstein, 1972, Okun et al., 2000, Meuwese et al., 2017). Evidence suggests the important role empathy plays in other kinds of socially undesirable behaviors such as violence, bullying, and sexual assault perpetration (Zych et al., 2019, Hudson-Flege et al., 2020). More recently research has found that lower empathy is associated with attention deficit and hyperactivity disorder and eating disorders (Kerr-Gaffney et al., 2019). As a protective asset, affective empathy has been shown to be tied to emotional regulation in social situations and cooperation toward collective goals (De Waal, 2008). Cognitive empathy has predicted higher friendship quality during adolescence (Chakrabarti and Baron-Cohen, 2006, Soenens et al., 2007, Knafo et al., 2008, Clark and Butler, 2020). Much research to date has focused on differentiating how dimensional factors that comprise empathy explain risk for psychiatric disorders including autism spectrum disorder, ADHD, callous unemotional traits, and aggressive behaviors (Kimonis et al., 2008, Moul et al., 2018, Georgiou et al., 2019, Milone et al., 2019, Martin-Key et al., 2020).

The dimensional structure of empathy may vary by gender. Research indicates that girls have shown higher total empathetic capacity than boys (Huang and Su, 2014, Lin and Chuang, 2018). In one study, as girls increased in age, affective and cognitive empathy increased, whereas in boys, affective, cognitive, and behavioral empathy were negatively associated with age (Overgaauw et al., 2017). Existing literature presents biological and environmental explanations for gender differences in empathy among adolescents. For example, research indicates that increased testosterone levels may explain empathetic capacity in boys (Hermans et al., 2006). Social construction of genders norms, and peer pressure to conform to these norms may influence expression of empathy in different contexts (Overgaauw et al., 2017). The potential for gender differences in empathy to be shaped by cultural and social expectations further supports the importance of assessing empathy measures and scale validity by gender and in different cultural contexts.

Additional research assessing differences in adolescents in individualist (Spain) and collectivist (Thailand) countries found that across both cultures, boys scored lower on affective and cognitive empathy than girls (Errasti et al., 2018). In a systematic review of empirical literature examining associations of empathy and prosocial measures in adolescents, 115 of 168 articles reviewed reported gender differences in empathy and/or prosocial behaviors, with 104 reporting higher levels of these measures in girls than boys (Silke et al., 2018). Most of the studies included in this review sampled adolescents from the United States, Italy, the Netherlands, and China, with almost no representation of African adolescents, and limited representation of adolescents from other collectivist cultures (Silke et al., 2018).

Cross-cultural validation of the EmQue-CA is needed to understand the factor-analytic structure of the measure in different contexts. Previous validation studies have demonstrated a three-factor structure among adolescents in China (Lin et al., 2021), Lithuania (Lazdauskas and Nasvytienë, 2021), and Portugal (da Silva et al., 2022). A valid cross-cultural measure of empathy is important because it allows for comparison of empathy across development and investigation of associations of empathy with mental health and wellbeing. Measurement of empathy in adolescents in contexts that are more collectivistic are needed because previous studies have shown that collectivistic countries have higher empathy than individualistic countries (Heinke and Louis, 2009, Chopik et al., 2017). Adolescents in collectivistic cultures may be socialized early in life in ways that are consistent with more interdependent contexts. Several studies have provided evidence suggesting prosocial behaviors by adolescents linked to empathy have significant association with peer norms and values, as well as cultural background and community context (Silke et al., 2018). A desire to adhere to social norms that promote empathy and associated behaviors may contribute to higher empathy in collectivist cultures compared to individualistic cultures.

A dimensional examination of empathy can help explain previously reported significant associations between empathy and mental health. A study of 17- to 25-year-olds by Caldwell-Harris and Ayçiçegi, 2006, found that individuals with extremely individualistic or collectivistic values that conflicted with the values of their communities were at higher risk of undesirable pathologies such as depression or social anxiety (Caldwell-Harris and Ayçiçegi, 2006). Particularly in collectivist cultures, high scores of individualism were positively associated with measures of paranoia, narcissism, antisocial and borderline personality disorders (Caldwell-Harris and Ayçiçegi, 2006). This suggests that research to further understand both cultural values and promotion of expressions of empathy and collectivist and individualistic behaviors; as well as relationships to mental health and wellbeing are important.

Existing literature demonstrates the important role that empathy places in social and emotional wellbeing, particularly in adolescence. Evidence of gender and cultural differences in empathy measures in children and adolescents highlights the importance of contextually relevant scale validation for measuring empathy. The objectives of this study are to validate the factor-analytic structure of empathy, and the reliability and validity of the EmQue-CA in a sample of Tanzanian adolescents and to test measurement invariance by gender.

## Materials and methods

### Sample selection

Participants in this study were recruited from the peri-urban Temeke Municipality in Dar es Salaam, Tanzania to participate in a three-arm comparative effectiveness trial Discover, an intervention to support social emotional mindsets and skills among very young adolescents (Cherewick et al., 2020). The Temeke Municipality includes urban and rural areas, with a diverse sociodemographic population from across Tanzania. According to The World Factbook, Dar es Salaam is the largest city in Tanzania with a rapidly growing population of approximately 7.4 million people (Central Intelligence Agency, 2022). The youth population in Tanzania is growing particularly fast, with 47% of the population under the age of 15 years and the total population of adolescents anticipated to double by 2055 (Cherewick et al., 2021). Although Tanzania has experienced a decline in poverty and rapid economic growth over the years, approximately 14 million people lived below Tanzania’s poverty line of TZS 49,320 (approximately USD 21) per adult equivalent in 2018 (World Bank, 2019). Especially in urban areas like Dar es Salaam, improvements to living conditions such as increased access to clean water and electricity significantly increased between 2012 and 2018, but human capital remained low (Human Capital Index in 2018 = 0.4; World Bank, 2019).

The Discover parent-study included demographic measures of the sample of very young adolescents participating in this study. The final sample of adolescents scored an average of 61.4 (*SD* 11.6) on the Tanzanian Poverty Scorecard, which ranges from 0-71 with higher scores indicating higher wealth (Cherewick et al., 2021). Participants also self-reported their general health on a scale of 1-4 with a sample mean of 1.94 (*SD* 0.98), where higher scores indicate better general health (Cherewick et al., 2021).

### Study procedures

Surveys took 45-60 min to complete. Participants were compensated for their time with a small gift of a notebook and pencil. These items were deemed to be of appropriate value at less than USD 2, after consulting with the research team, caregivers, teachers, and community members. A detailed study protocol of the parent study Discover Learning is provided elsewhere and describes participant recruitment and eligibility criteria (Cherewick et al., 2021). All parents/caregivers provided written consent and all adolescents provided verbal assent prior to administration of the survey questionnaire. These data were collected at baseline prior to the start of the intervention in June-July 2019. The analytic sample for this study was comprised of 579 adolescents (270 boys and 309 girls) ages 9-12 (mean age = 10.48; *SD* = 0.55).

### Ethics approval

This study was approved by the University of California Berkeley Committee for Protection of Human Subjects Institutional Review Board (IRB) - (CPHS Protocol Number: 2018-01-10628); in June 2018. The primary local partner in Tanzania, Health for a Prosperous Nation, obtained ethical clearance for these research activities from the National Institute of Medical Research – the local IRB in Tanzania (Ref. NIMR/HQ/R.8a/Vol. IX/2851) in August 2018.

### Survey measures

All participants completed a survey at baseline that included measures of demographic characteristics, social emotional skills and mindsets, and psychosocial assessment. Each measurement scale was selected based on previous use in low- and middle-income countries with adolescent populations. Items were adapted to reflect the study context and age of participants. All items in the survey were translated and back-translated from English to Swahili by Tanzanian research team members fluent in both languages. Swahili is Tanzania’s national language and lingua franca and is utilized in primary education settings during the first seven years of education. Survey translation was conducted to ensure items maintained their original meanings and were accurately understood by all participants.

### Empathy

Empathy was measured with the Empathy Questionnaire for Children and Adolescents (EmQue-CA) scale (Overgaauw et al., 2017). This scale has been validated with children and adolescents (10-15 years old) to assess affective empathy, cognitive empathy, and behavioral empathy. Response categories asked participants to rate on a 3-point scale whether the statement was 1 = “Not true”, 2 = “Somewhat true” or 3 = “True”. Affective empathy measures the extent to which adolescents feel the emotional state of the suffering person and includes six items (e.g., “When a friend is upset, I feel upset too,” “When a friend cries, I cry myself”). Cognitive empathy measures the extent to which the adolescent understands why another person is in distress and includes three items (e.g., “When a friend is angry, I tend to know why,” “If a friend cries, I often understand what has happened”). Five items measure behavioral empathy and the extent to which the adolescent is inclined to actively help or support the suffering person (e.g., “If a friend is sad, I like to comfort him/her,” “I want everyone to feel good”) (Overgaauw et al., 2017). Mean scores were calculated for each subscale such that higher scores reflect higher empathy. Previous validation of this scale has demonstrated adequate internal consistency (α = 0.70-0.74).

### Generosity

The Interpersonal Generosity Scale (IGS) contained 10 items to capture dimensions of generosity including attention, compassion, open-handedness, self-extension, courage, and verbal expression to assess concurrent validity with dimensions of empathy (Overgaauw et al., 2017). Responses were recorded on a Likert scale ranging from 1 = “Strongly disagree” to 4 = “Strongly agree.” Scores were aggregated by mean, with higher scores indicating greater generosity symptoms. Internal consistency for full generosity scale was α = 0.76 in this sample.

### African youth psychological assessment

The AYPA scale measures psychosocial health in African youth. The subscales included in this analysis include the subscale for prosocial and externalizing behaviors (Betancourt et al., 2014). Prosocial behavior measures were utilized to assess concurrent validity, and externalizing behaviors to assess convergent validity with dimensions of empathy. Scenario items were presented to the respondents who were asked to select how they would behave on a 4-point Likert scale (1 = “Never,” 2 = “Somewhat,” 3 = “Often,” 4 = “All the time”). Items from the prosocial subscale included statements such as “I listen to others and elders,” “I share with others,” and “I respect others.” Items from the externalizing behaviors subscale included statements like “I am disobedient,” “I insult friends,” and “I am a rough person.” Scores were aggregated by mean, with higher scores indicating greater measures of externalizing and prosocial symptoms. The internal consistency of the prosocial subscale was α = 0.70 and α = 0.77 for externalizing behaviors.

### Data analysis

Data was analyzed using Stata Statistical Software (StataCorp, 2017). The sample size for this analysis was adequate for completing exploratory factor analysis (EFA) and confirmatory factor analysis (CFA), following conventional requirements of 5-10 study subjects for each item analyzed, and meeting high subject to item ratios common in EFA of 20:1 (Tinsley and Tinsley, 1987, Osborne and Costello, 2009).

To establish concurrent validity, the African Youth Psychological Assessment (AYPA) prosocial subscale and the Interpersonal Generosity Scale (IGS) were used to assess correlation with the behavioral empathy dimension of the EmQue-CA. The intention to comfort factor has been used independently as an index of prosocial behavior in other studies (Blankenstein et al., 2020). The IGS measures six components of generosity: attention, compassion, open-handedness, self-extension, courage, and verbal expression. Each of the items on this scale have theoretical overlap with the item measures in the EmQue-CA. Positive associations between these measures and dimensions of empathy indicate acceptable concurrent validity and support the validity of the EmQue-CA scale in this population. Convergent validity of the empathy dimensions were assessed using the externalizing symptoms subscale of the AYPA. Negative associations between externalizing symptoms and dimensions of empathy indicate acceptable convergent validity and support the validity of the scale among Tanzanian adolescents. This study aims to test measurement invariance by gender in this sample. As previous studies have shown, empathy may have differential associations with various mental health measures by both gender and cultural context. Validation of the EmQue-CA to measure empathy in Tanzanian adolescent girls and boys may support opportunities for additional research on the relationships of empathy and mental health among adolescents in Tanzania and comparable contexts.

We first proceeded with EFA of the EmQue-CA by randomly splitting our sample in two. Principal component analysis results were assessed by using conventional criteria for EFA; eigenvalues ≥ 1.0, visual examination of the scree plot, factor loadings > 0.35, no cross loadings > 0.35, and uniqueness ≤ 0.80 on the first half of the analytical sample (*N* = 289). We used Oblimin factor rotation because factors have been shown to be correlated (Overgaauw et al., 2017, Lin et al., 2021). To assess the construct validity of the EmQue-CA, we conducted confirmatory factor analysis to test if a three-factor model fit these data on the second half of our sample. Goodness of fit was evaluated using the second half of the sample standard cut-off criteria including Bentler’s Comparative Fit Index (CFI); the Tucker Lewis Index (TLI) with values greater than 0.90; the Root Mean Square Error of Approximation (RMSEA) with values less than *p* = 0.08 and the Standardized Root Mean Error with values less than 0.08 (Tucker and Lewis, 1973, Steiger, 1980, Bentler, 1990, Hu and Bentler, 1999). Cronbach’s alpha and inter-item correlations were computed to assess reliability of constructs. This study used the conventional cut off standard of α ≥ 0.70 as criterion of acceptable reliability (Nunnally and Bernstein, 1978). To assess concurrent validity, we examined Pearson correlation coefficients between the empathy dimensions and subscale for prosocial behavior and generosity (Hill, 2009). To examine convergent validity, we examined the associations between the subscales of the EmQue-CA with the externalizing subscale from the AYPA. Concurrent validity was tested by examining the Pearson correlation coefficients between the subscales and prosocial behavior. Last, we tested for measurement invariance by gender including methods to assess configural invariance, metric invariance, scalar invariance, and residual invariance using the second half of the analytical sample (*N* = 290).

## Results

Results of the analyses determined that a three-factor structure of the EmQue-CA assessing cognitive, affective, and behavioral empathy (intent to comfort) is appropriate in very young Tanzanian adolescents from 9 to 12 years of age in low-resource settings. Reliability and validity were acceptable and similar to validations of the scale in other cultural contexts. Measurement invariance was not supported between genders. These key findings informed the validation and modification of the EmQue-CA for assessment of these domains of empathy in Tanzanian adolescents and comparable populations.

### Sample characteristics

Five hundred and seventy-nine (579) adolescents ages 9-12 were included in the analytic sample of this study (Table 1). The sample included 270 boys (46.6%) and 309 girls (53.4%). The mean age of study participants was 10.48 (*SD* = 0.55). 172 (29.7%) of participants were in 3rd grade, 261 (45.1%) in 4th grade and 146 (25.2%) in 5th grade. 345 (66.6%) of participants lived with both parents and 173 (33.4%) did not live with both parents. The average size of the household of study participants was 5.7 (*SD* = 2.6). Study participants reported a mean score of 61.4 (*SD* = 1.6) on the Tanzanian Poverty Scorecard (range 0-71).

**TABLE 1:** Sample characteristics (*N* = 579).

	*N*	%
**Gender**		
Boys	270	46.6
Girls	309	53.4
**Age**		
9	13	2.3
10	276	47.7
11	288	49.7
12	2	0.4
Mean Age (*SD*)	10.48 (0.55)	
**Grade**		
3	172	29.7
4	261	45.1
5	146	25.2
**Live with both parents**		
No	173	33.4
Yes	345	66.6
Household profile	Mean	*SD*
Household Size	5.7	2.6
Tanzanian Poverty Score (0-71)^1^	61.4	11.6

^1^Summative score of items 2-10 of the Tanzanian Poverty Scorecard; Higher score = higher wealth.

### Exploratory factor analysis

Exploratory factor analysis with oblique factor rotation and maximum likelihood (ML) estimation were used to examine the factor structure of the EmQue-CA from half of the baseline sample (*N* = 289, see Table 2). Principal component analysis results were assessed by using conventional criteria for EFA; eigenvalues ≥ 1.0, visual examination of the scree plot, factor loadings > 0.35, no cross loadings > 0.35, and uniqueness ≤ 0.80. These indices were used to determine a 3-factor solution best fit these data. Item 2, “I feel sad when I watch a sad movie” did not load on any factor > 0.30 and uniqueness > 0.80. For these reasons Item 2 was excluded from the subsequent confirmatory factor analysis.

**TABLE 2:** Oblique rotated factor loading from EFA on half of the analytic sample (*N* = 289).

Item	Statement	Affective	Cognitive	Behavioral	Uniqueness
Item 1	If my parent/guardian is happy, I also feel happy.	**0.44**	–0.01	0.07	0.78
Item 2	I often feel sad when I watch a sad movie.	0.33^2^	–0.10	0.12	0.85^1^
Item 3	When a friend is upset, I feel upset too.	**0.60**	0.15	–0.20	0.62
Item 4	When a friend cries, I cry myself.	**0.54**	0.06	–0.15	0.73
Item 5	If someone in my family is sad, I feel really bad.	**0.59**	–0.07	0.20	0.53
Item 6	I feel awful when two people quarrel.	**0.43**	–0.06	0.25	0.68
Item 7	When a friend is angry, I tend to know why.	–0.01	**0.87**	0.05	0.25
Item 8	If a friend is sad, I understand mostly why.	0.00	**0.89**	0.01	0.21
Item 9	If a friend cries, I often understand what has happened.	0.04	**0.80**	0.03	0.34
Item 10	If a friend is sad, I like to comfort him/her.	0.18	0.01	**0.48**	0.66
Item 11	I would like to help when a friend gets angry.	0.06	0.04	**0.61**	0.58
Item 12	If a friend has an argument, I try to help.	0.00	0.07	**0.71**	0.48
Item 13	I want everyone to feel good.	–0.11	0.01	**0.72**	0.53
Item 14	If a friend is sad, I want to do something to make it better.	–0.03	–0.02	**0.71**	0.52

^1^Uniqueness ≥ 0.80; ^2^Factor loading < 0.35; Factor loadings ¿ 0.35 are in bold.

### Confirmatory factor analysis and multi-group CFA for invariance by gender

**FIGURE 1:**
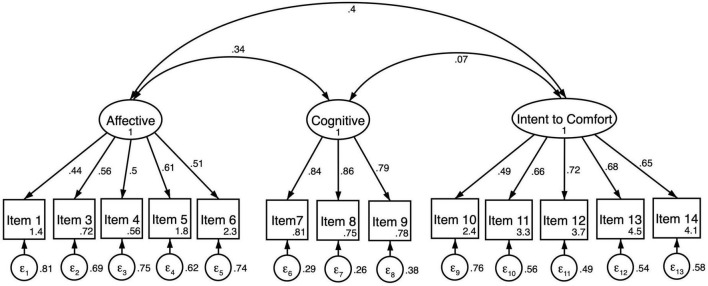
Three-factor confirmatory factor analysis of the EmQue-CA (13 items; *N* = 290). All factor loadings on latent factors significant at the *p* < 0.001 level. Values inside item boxes represent intercepts.

**FIGURE 2:**
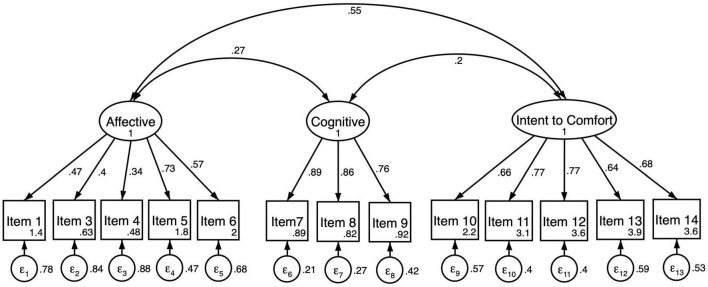
Three-factor confirmatory factory analysis of the EmQue-CA (13 items) for boys (*N* = 128). All factor loadings on latent factors significant at the *p* < 0.001 level. Values inside item boxes represent intercepts.

**FIGURE 3:**
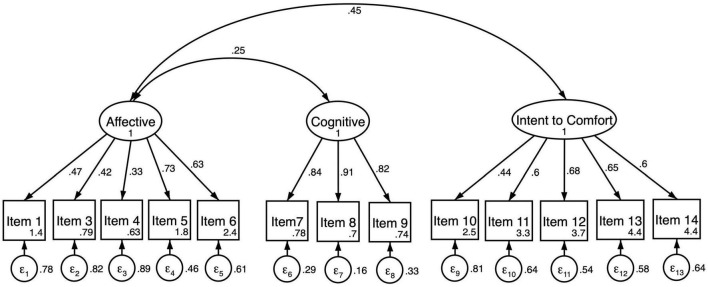
Revised three-factor confirmatory factory analysis of the EmQue-CA (13 items) for girls (*N* = 162). All factor loadings on latent factors significant at the *p* < 0.001 level. Values inside item boxes represent intercepts. Covariance path from cognitive empathy latent factor to intent to comfort latent factor is removed.

The three-factor model of EmQue-CA was tested using confirmatory factor analysis on the second half of the sample (*N* = 290, see Figure 1). The results for the second half of the analytical sample indicated adequate fit to these data evaluated against goodness of fit criterion shown in Table 3 (*CFI* = 0.92; *TLI* = 0.90; *RMSEA* = 0.062; *SRMR* = 0.070). Multigroup CFA was completed to test for measurement invariance by gender. Results indicated the measure failed tests of invariance by gender due to configural invariance (*SRMR* = 0.072 for girls; *SRMR* = 0.065 for boys). Covariance between cognitive and intent to comfort latent factors was significant for boys and shown in Table 4 (ρ*_*X,Y*_* = 0.149; *p* = 0.038) but not for girls (ρ*_*X,Y*_* = 0.002; *p* = 0.972). For these reasons a revised three-factor model was fit to the data for boys (Figure 2) and for girls (Figure 3). Results of the revised model improved model fit and met criterion of goodness of fit; *CFI* = 0.94; *TLI* = 0.92; *RMSEA* = 0.062; *SRMR* = 0.065 (Table 3).

**TABLE 3:** CFA fit indices for the total analytic sample and by gender (*N* = 579).

Sample	*χ ^2^*	*p-value*	*CFI*	*TLI*	*AIC*	*BIC*	*SRMR*	*RMSEA*
Total Sample	236.3	< 0.001	0.92	0.90	13057.2	13240.4	0.062	0.070
Boys	125.8	< 0.001	0.93	0.92	6110.6	6261.7	0.065	0.062
Girls	204.9	< 0.001	0.89	0.85	6941.8	7098.6	0.072	0.086
Girls-Revised CFA	136.3	< 0.001	0.94	0.92	6873.3	7029.9	0.065	0.062

CFI: Comparative Fit Index; TLI: Tucker Lewis Index; SRMR: Standardized Root Mean Square Residual; RMSEA: Root Mean Square Error of Approximation; AIC: Akaike’s Information Criteria; BIC: Bayesian Information Criteria.

**TABLE 4:** Covariance between affective, cognitive, and intent to comfort latent factors by gender.

Initial CFA Model				95 CI	95 CI
Covariances of latent factors	ρ *X,Y*	*SE*	*p-value*	Lower Bound	Upper Bound
**Cov(Affective, Cognitive)**					
Boys	0.429	0.08	< 0.001***	0.27	0.59
Girls	0.311	0.07	< 0.001***	0.18	0.44
**Cov(Affective, Intent to Comfort)**					
Boys	0.410	0.08	< 0.001***	0.25	0.57
Girls	0.389	0.08	< 0.001***	0.24	0.54
**Cov(Cognitive, Intent to Comfort)**					
Boys	0.149	0.07	0.038*	0.01	0.29
Girls	0.002	0.07	0.972	−0.13	0.14

*p ≤ 0.05; **p ≤ 0.01; ***p ≤ 0.001.

### Internal consistency

Reliability was assessed by evaluating inter-item covariance and Cronbach’s alpha. Reliability for the total empathy scale was acceptable (α = 0.75) and similar to other studies with adolescents including in China (α = 0.65), in Iran (α = 0.76-0.82), in Lithuania (α = 0.65-0.70), and Italy (α = 0.73-0.78) (Lazdauskas and Nasvytienë, 2021, Lin et al., 2021, Taheri et al., 2021, Liang et al., 2022). The affective empathy dimension demonstrated acceptable reliability (inter-item correlation = 0.16; α = 0.65), excellent reliability for cognitive empathy (inter-item correlation = 0.45; α = 0.89) and acceptable reliability for the behavioral empathy (intent to comfort) dimension (inter-item correlation = 0.10; α = 0.75).

### Concurrent validity

Concurrent validity was established using the prosocial subscale of the AYPA and the Interpersonal Generosity Scale. Affective empathy was associated positively with prosocial behavior (*r* = 0.23; *p* ≤ 0.001) and generosity (*r* = 0.19; *p* ≤ 0.001). Cognitive empathy was associated positively with generosity (*r* = 0.08; *p* = 0.045) but not with prosocial behavior (*r* = 0.08; *p* = 0.073). Behavioral empathy was associated positively with both prosocial behavior (*r* = 0.27; *p* ≤ 0.001) and generosity (*r* = 0.24; *p* ≤ 0.001). These results indicated good convergent validity of the EmQue-CA among Tanzanian adolescents.

### Convergent validity

Convergent validity was tested by assessing significant correlations between the EmQue-CA subscales and externalizing behaviors from the AYPA (Table 5). The behavioral dimension of empathy, intent to comfort, was significantly and negatively associated with externalizing behaviors (*r* = −0.18; *p* ≤ 0.001). Neither affective empathy (*r* = −0.01; *p* = 0.796) or cognitive empathy (*r* = 0.034; *p* = 0.412) was statistically significant in correlation with externalizing behaviors.

**TABLE 5:** Pearson correlation coefficients of EmQue-CA dimensions, and measures to evaluate concurrent and convergent validity.

	Concurrent Validity		Convergent Validity
EmQue-CA	Prosocial Behavior	Generosity	Externalizing Behaviors
Affective Empathy	0.23***	0.19***	−0.01
Cognitive Empathy	0.08	0.08*	−0.03
Intent to Comfort	0.27***	0.24***	−0.18***
Total Empathy	0.25***	0.22***	−0.07

*p ≤ 0.05; **p ≤ 0.01; ***p ≤ 0.001.

## Discussion

This study aimed to evaluate the psychometric properties of the EmQue-CA in a sample of Tanzanian adolescents. Exploratory and confirmatory factor analysis align with the factor structure of the EmQue-CA’s intended structure of the scale with the exclusion of Item 2. Item 2, “I often feel sad when I watch a sad movie” which falls in the affective empathy domain did not load on the affective empathy factor. It is plausible that this item did not resonate in this sample because adolescents are from a very low-resource context, where watching movies or having access to resources to watch movies are more limited than in high resource countries. Given these results, Item 2 may need to be adapted to the cultural context of settings, or a 13-item EmQue-CA scale may be more appropriate in future use with Tanzanian adolescents.

Analysis of the reliability (internal consistency) of factor dimensions was supported by our results. Convergent validity with prosocial behaviors was supported in this sample. Both affective and behavioral empathy subdimensions were significantly correlated with prosocial behaviors. Cognitive empathy was not associated with prosocial behavior, suggesting that capacity to intellectually understand another person’s distress does not necessarily motivate acting in a more prosocial way. These results align with previous research indicating a significant relationship between empathy and prosocial behaviors (Prot et al., 2014, Yu et al., 2020, Lin et al., 2021).

Convergent validity with externalizing behaviors was negatively associated with behavioral empathy, but not with affective or cognitive empathy, which underscores the importance of behavioral empathy in capturing a behavioral, affective-action dimension of empathy that the EmQue-CA was designed to measure. Behavioral empathy measures the tendency to help or support others through verbal and non-verbal behaviors that demonstrate affective and/or cognitive empathy (Tamayo et al., 2016, Clark and Butler, 2020). Previous studies have demonstrated the convergent validity of the EmQue-CA and negative correlation with callous unemotional traits and bullying and antisocial behaviors (Frick and White, 2008, Overgaauw et al., 2017, Liu et al., 2018, Zych et al., 2019, Lin et al., 2021). These measures were not available in this sample, however, future research should explore convergent validity of the EmQue-CA with these measures.

Measurement invariance by gender tests failed in this study due to configural invariance between girls and boys. Covariance between latent factors of cognitive empathy and behavioral empathy were significant for boys, but not for girls. The refitted measurement model omitting this path demonstrated excellent goodness of fit for girls. This finding suggests that boys may have capacity for cognitive and behavioral empathy that is acquired reciprocally between latent constructs, whereas for girls, the ability to practice empathy through behavioral action may be independent of capacity for cognitive empathy. While measurement invariance by gender was not supported, differences in latent construct associations by gender do not impact the validity of investigating dimensional structures of empathy using the scale. Instead these results suggest that the future research consider interpreting results by empathy dimension and gender to inform translation to potential adolescent programing.

Results in the current study confirm the validity of the EmQue-CA for adolescents in Tanzania and contributes to a growing body of research providing evidence of the cross-cultural relevance of measurement of behavioral empathy. These findings align with validation studies in Portugal and China, regarded as having similar collectivist social structures (Lin et al., 2021, da Silva et al., 2022) as well as among youth in Lithuania and adolescents primarily from the Netherlands, which are regarded as having more individualistic cultures (Overgaauw et al., 2017, Lazdauskas and Nasvytienë, 2021).

Given the highly significant concurrent and convergent associations of the behavioral empathy dimension, it is recommended that future studies, including qualitative research, explore how behavioral empathy is practiced in the Tanzanian context. Understanding culturally supported affective actions may leverage the protective and promotive associations of empathy on wellbeing. Previous studies have drawn connections between undesirable mental health pathologies (e.g., depression, anxiety) and having personal values in conflict with the values of their community (Caldwell-Harris and Ayçiçegi, 2006). Additional research should explore whether empathy mediates or moderates behavioral health outcomes including internalizing and externalizing symptoms and measures of wellbeing. Further research to understand and measure cultural values in conjunction with expressions of empathy, prosocial behaviors, and symptoms that are linked to pathologies like depression and anxiety (e.g., externalizing symptoms) has the potential to provide a more complete understanding of the relationship between mental health outcomes within the context of community expectations and social conditioning. This study demonstrates that the EmQue-CA is a valid measure of affective, behavioral and cognitive dimensions of empathy in a low-resource context and can be used to explore associations with psychopathology and wellbeing.

## Limitations

The EmQue-CA is a self-report measure, and it is possible participants were biased by social desirability factors. Measurement by caregivers or teachers could further support validation of the measure and dimensional structure. Tests of measurement invariance at different ages, including ages younger than 10 and older than 12, were not possible in this sample. However, a broader age range would help support findings of measurement invariance.

## Conclusion

These findings contribute to a growing body of research that suggests that behavioral empathy is an important dimension of measures of empathy and has differential associations with psychopathology across social and socioeconomic contexts. Empathy has also shown the potential to manifest differently among boys and girls during adolescence. It is important to consider the cultural and socioeconomic context, as well as the impact these cultural contexts may have on developmental expectations across genders during adolescent development before implementation of the EmQue-CA scale in new environments. Tests for measurement invariance by gender of the EmQue-CA failed due to configural invariance in covariance between latent constructs. Adjustment for this difference resulted in excellent model fit for girls, suggesting that gender specific factor scores of the EmQue-CA are a valuable measure of empathy among adolescents in Tanzania.

## Data availability statement

The raw data supporting the conclusions of this article will be made available by the authors, without undue reservation.

## Ethics statement

The studies involving human participants were reviewed and approved by University of California Berkeley Committee for Protection of Human Subjects Institutional Review Board and National Institute of Medical Research. Written informed consent to participate in this study was provided by the participants’ legal guardian/next of kin.

## Author contributions

MC and RD conceived and designed the original discover learning parent study. MC completed the development of survey instruments. MC, EH, and SS completed the data analysis and interpretation. MC, EH, RD, JL, and SS prepared the manuscript. All authors have contributed critically and significantly to drafting a final manuscript and approved the final version.

## Abbreviations

ADHD: Attention Deficit Hyperactivity Disorder
AIC: Akaike’s Information Criterion
AYPA: African Youth Psychological Assessment
BIC: Bayesian Information Criterion
CFI: Comparative Fit Index
EFA: Exploratory Factor Analysis
EmQue-CA: Empathy Questionnaire for Children and Adolescents
HPON: Health for a Prosperous Nation
IGS: Interpersonal Generosity Scale
NIMR: National Institute of Medical Research (Tanzania)
RMSEA: Root Mean Square Error of Approximation
SRMR: Standardized Root Mean Residual
TLI: Tucker-Lewis Index
TZS: Tanzanian Shilling
UC, Berkeley: University of California Berkeley.
